# Comparison between diffraction contrast tomography and high-energy diffraction microscopy on a slightly deformed aluminium alloy

**DOI:** 10.1107/S2052252515019995

**Published:** 2016-01-01

**Authors:** Loïc Renversade, Romain Quey, Wolfgang Ludwig, David Menasche, Siddharth Maddali, Robert M. Suter, András Borbély

**Affiliations:** a École des Mines de Saint-Étienne, CNRS UMR 5307, 158 cours Fauriel, 42023, Saint-Étienne, Cedex 2 France; b European Synchrotron Radiation Facility (ESRF), 6 rue Jules Horowitz BP 220, 38043 Grenoble, France; c Department of Physics, Carnegie Mellon University, 5000 Forbes Avenue, Pittsburgh, PA 15213, USA

**Keywords:** X-ray diffraction, three-dimensional X-ray diffraction (3DXRD), DCT, HEDM, image registration, polycrystal plasticity

## Abstract

The grain structure of an Al–0.3 wt%Mn alloy deformed to 1% strain was reconstructed using diffraction contrast tomography (DCT) and high-energy diffraction microscopy (HEDM). 14 equally spaced HEDM layers were acquired and their exact location within the DCT volume was determined using a generic algorithm minimizing a function of the local disorientations between the two data sets. The microstructures were then compared in terms of the mean crystal orientations and shapes of the grains. The comparison shows that DCT can detect subgrain boundaries with disorientations as low as 1° and that HEDM and DCT grain boundaries are on average 4 µm apart from each other. The results are important for studies targeting the determination of grain volume. For the case of a polycrystal with an average grain size of about 100 µm, a relative deviation of about ≤10% was found between the two techniques.

## Abstract 2

The grain structure of a slightly deformed Al–0.3 wt%Mn alloy was reconstructed using diffraction contrast tomography (DCT) and high-energy diffraction microscopy (HEDM). The direct comparison shows that DCT can detect subgrain boundaries with disorientations as low as 1° and that HEDM and DCT grain boundaries are on average 4 µm apart from each other.

## 1. Introduction   

Developments in high-energy synchrotron radiation sources during the last 15 years have paved the way for novel diffraction-based imaging techniques. Most of them are labelled as three-dimensional X-ray diffraction (3DXRD) (Poulsen, 2012 ▸) and they offer fast and non-destructive access to the granular structure of polycrystalline materials. 3DXRD usually refers to a set of techniques sharing common core features, such as the use of hard X-rays, tomography-type sample scanning, and the application of indexing and reconstruction algorithms. All the methods allow the characterization of the position, size and crystallographic orientation of single grains embedded in the bulk of the polycrystal. Based on the spatial resolution of the detectors involved, these techniques can be categorized as *near-field* and *far-field* methods (Poulsen, 2012 ▸). The former use detectors with a pixel size of a few micrometres and are capable of imaging spatially resolved grain morphologies (Suter *et al.*, 2006 ▸; Lauridsen & Schmidt, 2001 ▸; Ludwig, King *et al.*, 2009 ▸). On the other hand, far-field methods using detectors with large pixels (50–200 µm) enable the determination of average properties only, such as the mean grain orientation, the grain volume or the grain-average strain tensor (Margulies *et al.*, 2002 ▸; Martins *et al.*, 2004 ▸; Oddershede *et al.*, 2010 ▸ *et al.*, 2011 ▸; Borbély *et al.*, 2014 ▸).

Today, 3DXRD techniques have reached maturity and contribute more and more to a paradigm shift in materials science regarding microstructure characterization. Until very recently, checking the accuracy of reconstructed structures was done against data acquired with destructive laboratory techniques, especially by electron back-scatter diffraction (EBSD). However, due to the proliferation of diffraction microscopy methods, a direct comparison between them is now possible. An initial example is the work of Nervo *et al.* (2014 ▸) comparing far-field 3DXRD (Poulsen, 2004 ▸, 2012 ▸) with DCT (Johnson *et al.*, 2008 ▸; Ludwig, King *et al.*, 2009 ▸) as a near-field technique. This allowed assessment of the accuracy of the indexing algorithms in terms of grain orientation, volume and centre-of-mass position. It was shown that the characterization of crystallographic orientation and grain size is quite reliable, but significant differences were found in the retrieved grain positions. The latter disagreement was related to the difference in pixel size of the far-field (48.5 µm) and near-field detectors (1.4 µm). The comparison also showed the presence of several unmatched grains attributed to the indexing routines and sensitive to low completeness and reduced counting statistics, which is typical for small grains (generally smaller than half the pixel size of the far-field detector).

After this first assessment of the average structural parameters, it is interesting to ask if the comparison between 3DXRD methods can be further extended towards higher spatial resolutions, for example concerning grain morphology. Four diffraction imaging methods can be identified for this purpose: (i) the previously mentioned DCT, (ii) HEDM (Suter *et al.*, 2006 ▸), (iii) scanning 3DXRD (S3DXRD) (Hayashi *et al.*, 2015 ▸) and (iv) differential-aperture X-ray microscopy (DAXM) (Larson *et al.*, 2002 ▸). Most of these techniques use monochromatic beams of different geometries, a broad box beam in the case of DCT, a planar beam for HEDM and a pencil beam for S3DXRD. DAXM uses a polychromatic micro-focused pencil beam. Pencil beams are well suited for local analysis on the micrometre scale (Larson & Levine, 2013 ▸), while larger beams are better adapted for characterizing millimetre-sized samples.

Due to the planar beam involved, HEDM uses a layer-by-layer approach to build up three-dimensional representative structures. The robustness of the method has already been tested on plastically deformed materials showing intragranular orientation spread (Hefferan *et al.*, 2012 ▸; Li *et al.*, 2012 ▸; Li & Suter, 2013 ▸; Spear *et al.*, 2014 ▸; Pokharel *et al.*, 2015 ▸). These high-quality results are somewhat counterbalanced by the relatively long analysis and scanning time required to build up representative volumes. Conversely, using a wide box beam and a tomography-type reconstruction algorithm, DCT excels with shorter scanning and analysis times, but at the cost of reduced structural information. The reconstruction unit in DCT is the grain/subgrain and as such is much larger than the pixel size utilized in HEDM. This feature assigns a constant crystallographic orientation to each unit, which limits the application of DCT to microstructures with small orientation gradients (Ludwig, King *et al.*, 2009 ▸; King *et al.*, 2008 ▸; Herbig *et al.*, 2011 ▸). However, there is much interest in reducing this reconstruction unit and extending the application of the method to plastically deformed crystals, an aspect which also motivated the present investigations. By choosing a slightly deformed polycrystal (of 1% tensile strain), the comparison between DCT and HEDM can therefore answer two questions related to the goodness of their reconstructions and the applicability of the current DCT code to slightly deformed crystals.

Since the pixel sizes of the detectors used with HEDM and DCT are nearly identical, one expects a close spatial resolution of their final reconstructions, which leads to a very challenging comparison regarding, for example, grain shape. DCT reconstructions have already been checked against both phase contrast tomography (Ludwig, Reischig *et al.*, 2009 ▸) and EBSD mapping (Johnson *et al.*, 2008 ▸; Syha *et al.*, 2013 ▸). However, both comparisons lack completeness, since crystallographic information is missing in phase-contrast tomography and the EBSD scan only has reduced spatial information (a slice in the three-dimensional structure). To overcome these limitations, the present work uses several HEDM ‘slices’ and aims at a six-dimensional confrontation and cross-validation of the two methods. For the sake of completeness, comparisons with EBSD and far-field 3DXRD will also be presented.

## 2. Experimental procedure and reconstructions   

### 2.1. Sample preparation and mounting   

**Figure 1:**
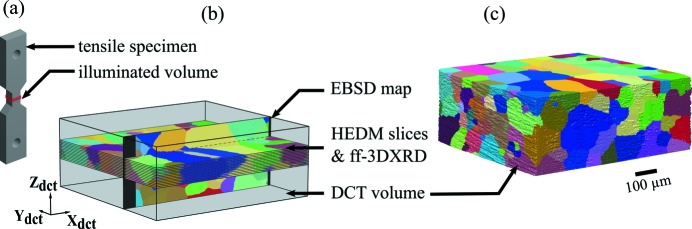
Sample and grain structure imaged with DCT, HEDM and EBSD. (*a*) The dog-bone shaped specimen, with a height of 14 mm and a gauge length of 1.5 mm. The 350 µm-high illuminated volume is shown in red. (*b*) The positions of the HEDM and EBSD slices in the DCT volume. Black margins on the EBSD map indicate regions removed by electro-polishing. (*c*) The DCT volume, which is about 350 µm high. The colour coding is according to equation (6).

A dog-bone shaped tensile specimen made of a high-purity Al–0.3 wt%Mn alloy cold rolled at 80% was prepared by electro-discharge machining. Its geometry, shown in Fig. 1 ▸, presents a reduced square section of 1 mm^2^ and a gauge length of 1.5 mm. The sample was recrystallized at 450°C for 20 min, resulting in an average grain size of about 100 µm. To test the applicability of DCT to deformed structures, the specimen was deformed in tension up to a strain of 1%. For scanning, the sample was mounted on a rotation stage and aligned with its deformation axis parallel to the rotation axis of the gonio­meter. The DCT and HEDM scans were performed consecutively on beamline ID11 at ESRF.

### 2.2. Diffraction contrast tomography   

The DCT scan was done using a monochromatic wide beam with a cross-section of 1.0 × 0.350 mm and an energy of 41.7 keV. Diffraction images were recorded with a FReLoN 2k x 2k X-ray detector with an effective pixel size of 1.4 µm positioned normal to the incident beam at a sample-to-detector distance of 7 mm. A full 360° scan was performed with an angular integration step of 0.1° and an exposure time of 2 s, which resulted in a total of 3600 images and a scan duration of 2 h. The large field of view of the detector permitted simultaneous recording of both the transmitted and diffracted beams. Data were analysed with the DCT software (http://sourceforge.net/projects/dct) available at the beamline. The diffraction spots were used to reconstruct the grains, while the direct transmitted intensity permitted the tomographic reconstruction of the sample shape, which was used as a mask for the grain map (Ludwig, Reischig *et al.*, 2009 ▸; Reischig *et al.*, 2013 ▸). The resulting microstructure (Fig. 1 ▸ *c*) is described on a cubic grid with a voxel size of 1.4 µm and contains about 400 grains.

### 2.3. High-energy diffraction microscopy and far-field 3DXRD   

The measurements were taken at an energy of 60 keV using a line-focused beam of height 2 µm and width 1.5 mm. HEDM data were acquired simultaneously with a ‘so-called’ three-dimensional detector (Poulsen *et al.*, 2010 ▸) made of two semi-transparent 2k x 2k detectors with effective pixel sizes of 1.5 and 4.5 µm and placed at sample-to-detector distances of 5 and 15 mm, respectively. Contrary to the usual HEDM setup, where the direct beam hits the detector near its bottom edge (Suter *et al.*, 2006 ▸), the beam centre was positioned near the centre of the three-dimensional detector. Thanks to the semi-transparency of the three-dimensional detector, a third FReLoN X-ray camera with a pixel size of 47.5 µm could be positioned in the far-field region at a distance of 250 mm, allowing simultaneous collection of far-field diffraction images, which were analysed and compared with the HEDM results.

HEDM data collection consisted of measuring 14 two-dimensional cross-sections near the centre of the DCT volume. The layers were displaced uniformly along the vertical axis by a distance of about 6 µm. Imposing a sample rotation of 180° and an integration step size of 0.25°, 720 images were acquired simultaneously with all three detectors for each layer. The exposure time was 2 s, resulting in a total measurement time of 5.5 h. HEDM reconstructions were performed with the forward-modelling software *IceNine* (Suter *et al.*, 2006 ▸; Li & Suter, 2013 ▸). For each of the 14 layers, a sample space centred on the rotation axis and larger than the illuminated region was chosen and meshed with around 390 000 equilateral triangles. Their edge length of 2.5 µm was chosen as a compromise between the pixel sizes of the two semi-transparent detectors which limit the spatial resolution of the reconstruction. For each triangular element, the crystal orientation was determined by Monte Carlo optimization of the overlap between the simulated and experimental diffraction spots. The reconstruction software provided maps of the confidence metric, defined as the fraction of simulated peaks that overlap measured peaks on the two near-field detectors. A confidence of 1 corresponds here to about 50 matching peaks. Only elements with a confidence above 0.2 were kept in the final grain maps. The average confidence was around 0.5 with a maximum of 0.68. These values are somewhat lower than usual, which can be explained by a lower signal-to-noise ratio in the images measured by the semi-transparent detectors and a slight misalignment of the rotation axis relative to the plane normal of the line-focused beam.

Far-field measurements were analysed using in-house software, following standard procedures for image analysis and indexing (Kenesei, 2010 ▸; Moscicki *et al.*, 2009 ▸). The results provided, for each grain in each layer, a centre-of-mass position, an average size and an average crystallographic orientation.

### 2.4. Electron back-scatter diffraction   

Faced with the difficulty of obtaining an EBSD map in the 80 µm-thick volume superimposed with one of the HEDM layers, the observation was instead performed on a section parallel to the tensile axis. The sample surface was prepared by mechanical grinding, polishing with diamond suspensions of grain sizes 3 and 1 µm, and finally electro-polishing with a commercial electrolyte (Struers, AII). EBSD characterization was performed in a Zeiss Supra 55VP scanning electron microscope (SEM) equipped with a high-resolution NordlysNano camera (Oxford Instruments) and *AZtecHKL* software suite, using an acceleration voltage of 20 keV, a sample tilt of 70° and a working distance of 15 mm. The scan was centred at the middle of the gauge length and covered a region of 450 µm in height and 1 mm in length with a step size of 3 µm. Fig. 1 ▸(*b*) illustrates the positions and orientations of the different HEDM and EBSD sections with respect to the DCT volume and the sample. To provide reference intragranular orientation distributions, separate EBSD observations were made under identical conditions on a non-deformed material.

## 3. Registration   

A direct unbiased comparison between the DCT, HEDM and EBSD microstructures can only be carried out if their respective locations are known accurately. The present experiment involves uncertainties associated with the vertical focusing of the beam between the DCT and HEDM measurements, the use of different detectors, and the mechanical preparation of the EBSD observation surface. While the relative positions of selected data sets can be estimated by simple visual inspection, an automated registration method is required to refine them.

Registration between two data sets, which consisted here of determining the position of the first (HEDM or EBSD) with respect to the second (DCT), was done by maximizing the correspondence between the two, while accounting for potential distortions. This was carried out on a region located at the intersection between the DCT and HEDM (or EBSD) data sets, and referred to as the registration window. For simplicity, HEDM and EBSD data were remapped onto a grid of square pixels of the same size as in DCT (1.4 µm). The registration approach used here is based on a method developed previously for a comparison between X-ray nanotomography and SEM grey-level images (Quey *et al.*, 2013 ▸), and therefore needs to be extended to the more delicate case of crystal orientation data.

Let us consider a pixel *p* in the HEDM slice that is being registered. Its position is referred to as 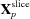 in the coordinate system of the slice and 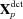 in the coordinate system of the DCT volume. The geometric operator that relates 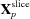 and 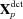 is an affine transformation that can be expressed as
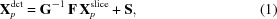
where **F** combines scaling and shearing, **G** is a rotation and **S** a shift vector. **F** can be written as
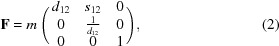
where *m* is a magnification factor, *d*_12_ is a scaling ratio between directions 1 and 2 of the slice, and *s*_12_ is the in-plane shear. The orientation of the slice coordinate system with respect to the DCT frame is given by the orthogonal matrix **G**. Conversely to the approach of grey-level registration, rotating the HEDM slice implies a change of the reference coordinate system in which the local orientations are expressed. Describing the crystallographic orientation of a pixel with the matrices 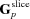 and 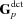 in the slice and DCT frames, respectively, the frame transformation can be written as
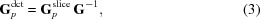
For each pixel *p* in the slice image, 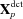 and 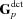 are obtained using equations (1) and (3). The local crystallographic orientation in the DCT volume at position 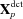 is referred to as 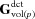 and is approximated by the orientation of the corresponding voxel. The disorientation angle between 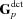 and 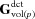 is defined as the smallest misorientation angle out of all symmetrically equivalent orientations, which implies the use of crystal symmetry operators (24 for cubic crystals). The disorientation angle associated with pixel *p* is referred to as θ_*p*_.

The registration problem then consists of finding the values of **F**, **G** and **S** in equations (1) and (3) that yield the best possible match between the slice and the DCT volume. This is quantified using the following function
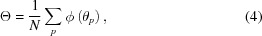
where *N* is the number of pixels that fall in the registration window and ϕ is a weighting function chosen so as to reduce the influence of high disorientation angles compared with lower ones. This can be achieved by using an exponential function
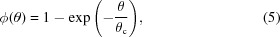
where θ_c_ is a constant reference angle. ϕ(θ) is equal to zero for θ = 0 and reaches 1 for θ > 5θ_c_. The lower the value of Θ, the better the match between the slice and its intersection with the DCT volume. The orientation matrix **G** can be described by three independent variables, *R*_1_, *R*_2_ and *R*_3_, when using the Rodrigues vector parametrization (Frank, 1988 ▸). Equations (1)–(5) then form a nonlinear optimization problem with Θ as the objective function, which is minimized over the set of nine unknown geometric variables (*m*, *d*_12_, *s*_12_, *R*_1_, *R*_2_, *R*_3_ and the shift vector components *s_x_*, *s_y_* and *s_z_*). The minimization was performed using the *NLOPT* library for local derivative-free optimization (Rowan, 1990 ▸; Johnson *et al.*, 2008 ▸) and the *ORILIB* library for orientation and rotation calculations (Quey, 2012 ▸).

For registering HEDM slices, only the magnification factor *m*, the three orientation components (*R*_1_, *R*_2_, *R*_3_) and the coordinates (*s_x_*, *s_y_*, *s_z_*) of the translation were refined. The scaling ratio *d*_12_ and the in-plane shear *s*_12_ were kept at constant values of 1 and 0, respectively. The initial value of *s_z_* was estimated by visual inspection of the DCT volume, while the other parameters were set to default values (*m* = 1, *s_x_* = 0, *s_y_* = 0, *R*_1_ = 0, *R*_2_ = 0, *R*_3_ = 0). Registration was repeated independently for each HEDM slice. This resulted in a rotation of the slices by about 0.66 ± 0.01°, an *xy* translation of a few pixels and a magnification factor *m* = 0.996 ± 0.003. The refined *z* coordinates of the slices show a uniform vertical distribution with an average spacing of 5.8 ± 0.15 µm, in agreement with the imposed *z* displacement. The error in the fit parameters represents the standard deviation over the 14 registered slices.

The same procedure was also applied to register the EBSD map with the DCT volume, but this time the scaling *d*_12_ and the shear *s*_12_ were also refined. Initially, the EBSD frame was positioned parallel to the *xz* plane (Fig. 1 ▸), which corresponds to *R*_1_ = 1, *R*_2_ = 1 and *R*_3_ = −1. The initial values of the translation components were estimated by visual inspection of the DCT volume, while the other parameters were set to default values (*m* = 1, *d*_12_ = 1, *s*_12_ = 0). The optimization resulted in a rotation of the EBSD map by about 4.4° and a magnification of 0.98. The distortion factors *d*_12_ and *s*_12_ were about 0.99 and 0.01, respectively.

## 4. Results   

The geometric parameters refined by the registration procedure were used to extract cross-sections of the DCT volume for comparison with HEDM and EBSD. These maps should be the best suitable for an unbiased comparison between the techniques regarding grain shape and crystallographic orientation.

### 4.1. Comparison between DCT and HEDM   

**Figure 2:**
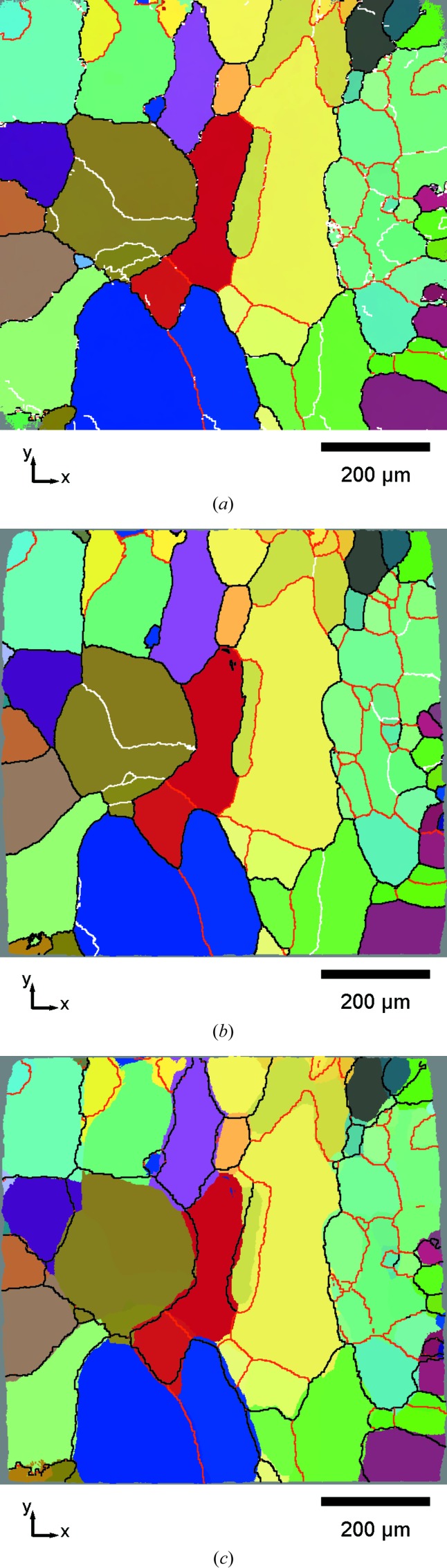
Comparison of registered DCT and HEDM grain structures. (*a*) An HEDM reconstruction of the eighth slice, (*b*) the registered DCT cross-section, (*c*) a DCT map with superimposed HEDM grain boundaries. Boundaries with different disorientations (δ) are indicated in white (1° ≤ δ ≤ 5°), red (5° ≤ δ ≤ 15°) and black (δ ≥ 15°). The pixel size is 1.4 µm. Axes labels *x* and *y* refer to the slice coordinate system. The colour coding is according to equation (6).

Fig. 2 ▸ shows a comparison between HEDM and DCT microstructures for a single slice. Crystal orientation is represented in a false colour scheme by relating the RGB colour components to Rodrigues vector components *R_i_*, according to the following scheme
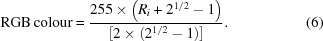
The superposition of the DCT orientation map and the HEDM grain boundaries is shown in Fig. 2 ▸(*c*). The cross-section of the DCT volume contains about 95 individual grains with an average size of about 100 µm. High-angle boundaries in black (≥ 15°) separate regions of visibly distinct colours, while moderate-angle boundaries in red (≥ 5°) generally help to distinguish areas of similar colour. Low-angle boundaries in white (≥ 1°) are also drawn in Figs. 2 ▸(*a*) and 2 ▸(*b*) to emphasize the subgrain structures caught by both HEDM and DCT.

**Figure 3:**
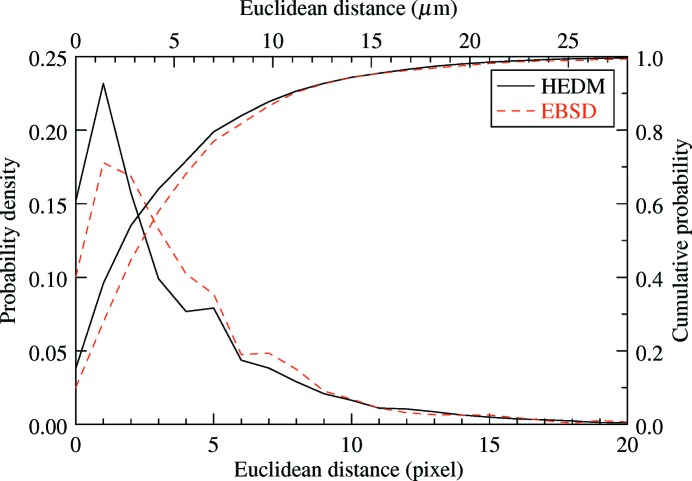
The probability density and cumulative probability of the Euclidean distance between DCT and HEDM/EBSD grain boundaries. The HEDM results are for all 14 slices. The pixel size is 1.4 µm. The mean values are 3.1 pixels (4.3 µm) for HEDM and 4.0 pixels (5.6 µm) for EBSD.

A very good overall agreement between reconstructions can be observed. To quantify this similarity in terms of spatial resolution and grain morphology, individual grains were identified in the HEDM maps and matched with DCT grains. The structure shown in Fig. 2 ▸ contains 80 unequivocally matched grains, a number which increased to 149 after gathering the results for all 14 slices. Based on this matching, the overlap between HEDM and DCT grains could be identified by counting the DCT pixels located in ‘neighbouring’ grains on the HEDM map, or *vice versa*. This number of erroneous pixels was divided by the total number of pixels in the registration window, which resulted in an overlap ratio of 87%. Additionally, Euclidean distance mapping was applied to measure the two-dimensional distance between the grain boundaries (only for high- and moderate-angle types) of the HEDM slices and corresponding DCT cross-sections. For each pixel of the DCT boundary network, the distance to the nearest HEDM boundary was calculated, which resulted in the statistics shown in Fig. 3 ▸. The distribution exhibits a maximum at a distance of 1 pixel (1.4 µm) and with an average of around 3 pixels, which is about 4% of the average grain size. The cumulative distribution function also shows that 50% of the DCT boundary pixels are at most 2 pixels away from the HEDM boundaries, within the reconstruction resolution. Considering the 2.5 µm triangles used in the HEDM reconstructions, this agreement can be considered excellent.

**Figure 4:**
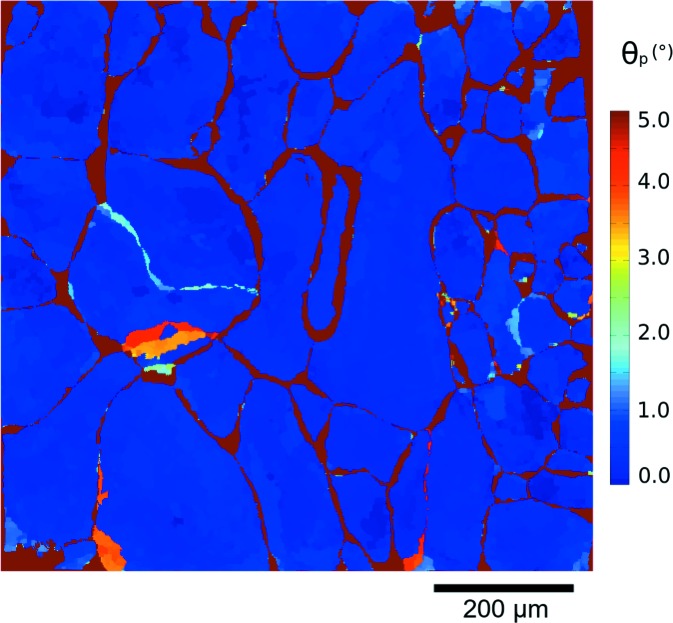
Residual disorientation map between HEDM and DCT after registering. The map corresponds to the eighth HEDM slice, shown in Fig. 2 ▸.

**Figure 5:**
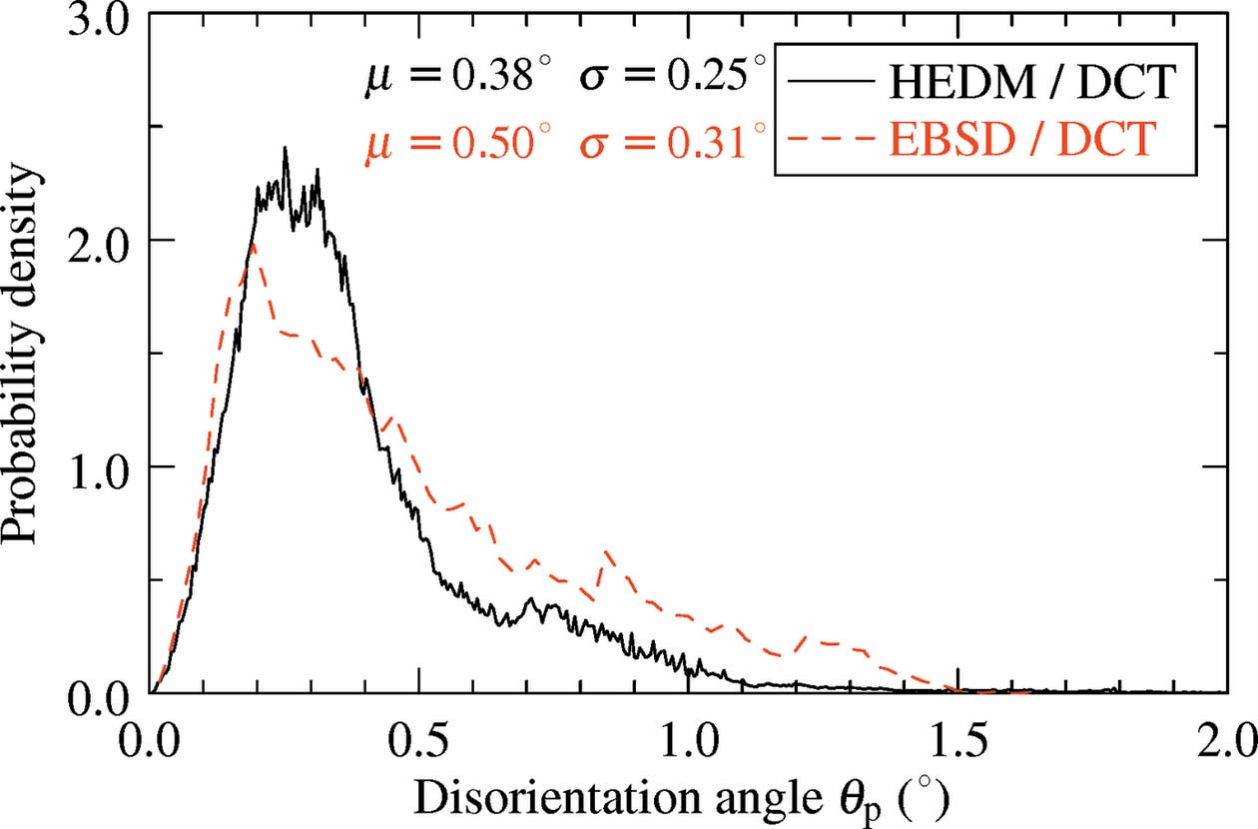
The distribution of residual disorientation angles θ_*p*_ with respect to DCT after registration of HEDM and EBSD maps. Only values for overlapping regions with θ_*p*_ ≤ 2° were considered.

This registration procedure, based on the minimization of local disorientation, also provides as its result the local residual disorientation θ_*p*_ between the two reconstructions. This map, considered as a rough comparator of the crystallographic orientations given by the two techniques, is shown in Fig. 4 ▸ (corresponding data in Fig. 2 ▸). Moderate and highly dis­oriented regions appear in red and illustrate the distance between DCT and HEDM grain boundaries. These zones contrast with blue regions characterized by low disorientations, which can be regarded as the overlapping grain regions as described previously. The disorientation map shows that the residual θ_*p*_ in the overlapping region is typically less than 1°. Colour variation is due to intragranular orientation variation in the HEDM maps compared with the constant grain orientation of DCT. Fig. 5 ▸ shows the distribution of θ_*p*_ over the blue overlapping regions (for all 14 HEDM slices) and has a maximum at about 0.3°, a mean of 0.38° and a standard deviation of 0.25°.

### 4.2. Comparison between DCT and EBSD   

**Figure 6:**
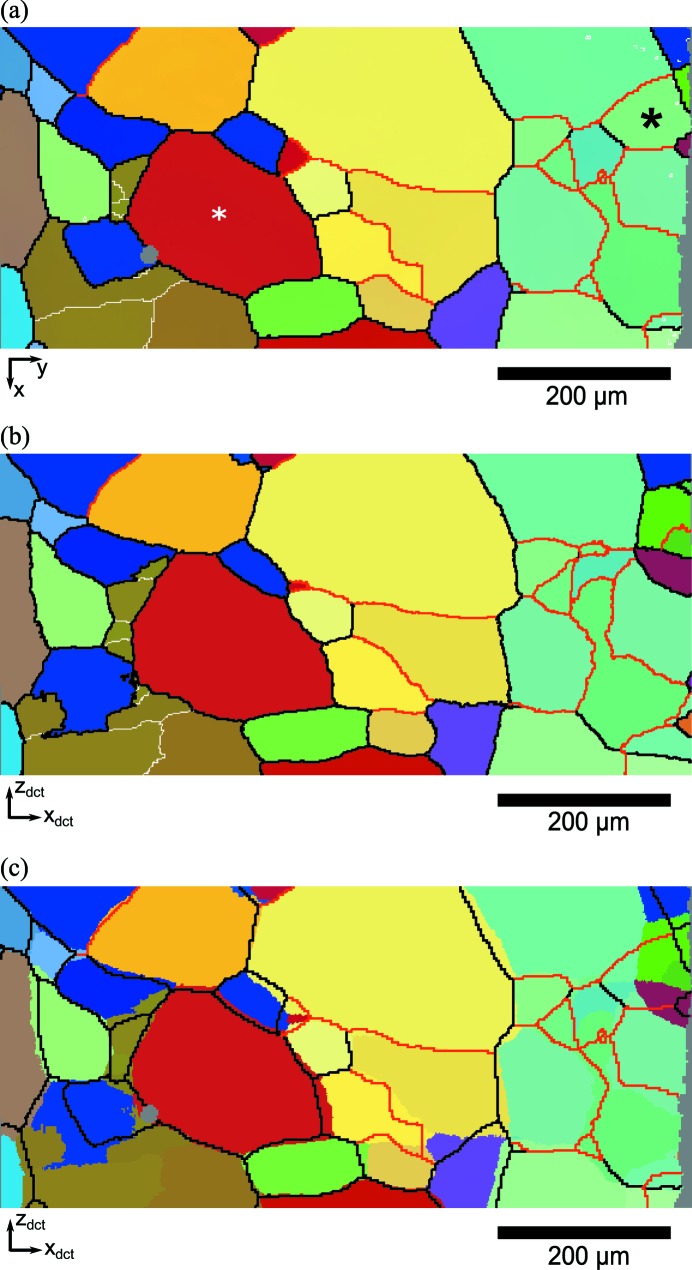
Comparison of grain structures revealed by DCT and EBSD on the same slice after registration [colour coding according to equation (6)]. (*a*) The EBSD orientation map, (*b*) the DCT cross-section obtained from registration, (*c*) the DCT map with superimposed EBSD grain boundaries (white: 1° < δ < 5°; red: 5° < δ < 15°; black: δ > 15°). The pixel size is 1.4 µm. The **x** and **y** unit vectors refer to the EBSD coordinate system, which has been flipped for consistency with Fig. 1 ▸. In part (*a*), the white asterisk marks the grain analysed in Fig. 7 ▸(*b*) and the black asterisk marks the grain which is missing in DCT.

The EBSD grain map shown in Fig. 6 ▸(*a*) contains 45 grains, which have an average size of about 80 µm. The corresponding DCT cross-section is shown in Fig. 6 ▸(*b*). Crystallographic orientations are expressed in the DCT reference frame and coloured using the scheme defined by equation (6). The angle between **Z**_ebsd_ and **Y**_dct_, which is not visible in the figure, is about 2.4°, meaning that the difference in *Y*_dct_ coordinate from one side of the map to the other can reach a few dozens of pixels.

Fig. 6 ▸(*c*) shows the extracted DCT cross-section, together with the grain boundaries obtained from EBSD. A good overall agreement is observed. The overlap ratio between the two grain structures (of 82%) is lower than that between DCT and HEDM, which is partly due to a grain present in the EBSD image (marked by a black asterisk) but not identified by DCT. The failure of the DCT code to index the grain is related to the close orientation of surrounding grains (blue region in Figs. 2 ▸ and 6 ▸), which led to a too small number of identified Friedel pairs (below the imposed limit of four). However, performing another registration on only the left-hand side of the EBSD map did not significantly change the position of the slice, indicating that the influence of the missing grain on the present results is negligible. The distribution of interboundary distances is shown in Fig. 3 ▸, together with the corresponding results of the DCT–HEDM comparison. The two results are quite similar, in that both probability density distributions have a maximum at about 1 pixel, but the average interboundary distance for the DCT–EBSD comparison is slightly larger (about 4 pixels, compared with the value of 3 pixels for DCT–HEDM). Relating the registration residuals for HEDM and EBSD comparisons (Fig. 5 ▸) leads to a similar statement, namely that the distribution of θ_*p*_ in the case of EBSD is comparable with that for HEDM but exhibits a larger spread with a higher and longer tail. These results suggest a better statistical agreement between HEDM and DCT maps, which is probably related to the limited statistics available in a single EBSD image.

**Figure 7:**
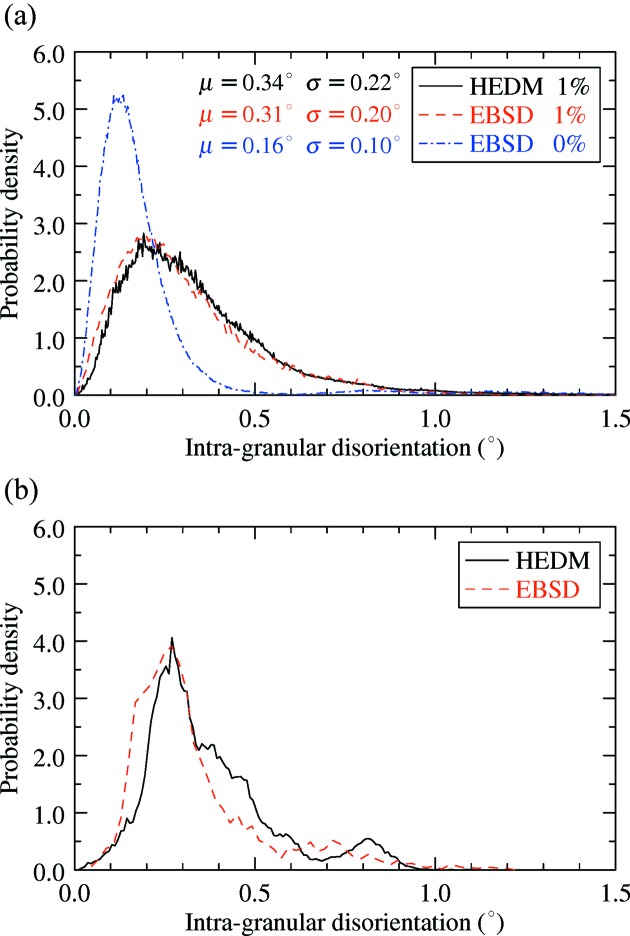
Intragranular disorientation distributions revealed by HEDM and EBSD, for (*a*) the full data sets and (*b*) the single grain marked by a white asterisk in Figs. 6 ▸(*a*) and 8 ▸.

The available HEDM and EBSD reconstructions made possible a third comparison. The orientation spread of single grains was quantified and compared between the analysed deformed sample and an undeformed specimen, by computing the intragranular disorientation metric (IGD) defined for each pixel as the disorientation angle between the orientation of the pixel and the average orientation of the grain to which it belongs. The distribution of IGD is given in Fig. 7 ▸(*a*) for the full data sets and in Fig. 7 ▸(*b*) for a single grain present in the HEDM and EBSD maps. The comparison shows excellent agreement between HEDM and EBSD for the full maps characterizing the deformed sample (Fig. 7 ▸ *a*), when the two distributions superimpose. Good agreement was also found in the case when only the IGDs for a single grain were compared (Fig. 7 ▸ *b*), although the two curves do deviate more from each other, probably due to the reduced number of data points. Further comparison between deformed and undeformed samples is shown in Fig. 7 ▸(*a*). The latter distribution has a maximum at about 0.12°. The mean and standard deviation are half the values characterizing the deformed sample (both EBSD and HEDM), indicating an increased intragranular orientation spread even at only 1% plastic deformation.

### 4.3. Comparison between HEDM and far-field 3DXRD   

**Figure 8:**
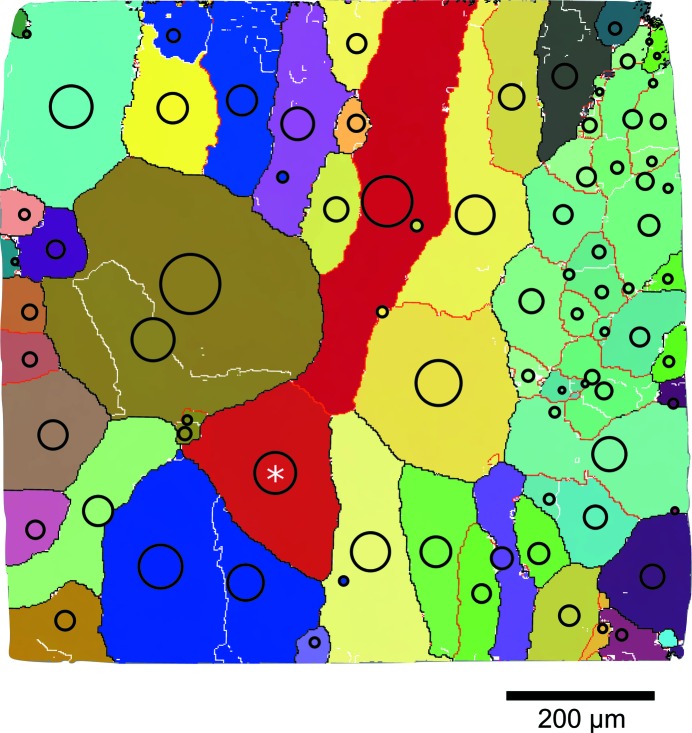
Comparison of far-field 3DXRD results with the HEDM map of layer No. 3. 81 grains obtained from far-field data are represented as black circles centred on the estimated grain centre-of-mass position; the radii are proportional to the grain sizes obtained from far-field intensities (downscaled for the sake of visibility). Only a few differences can be observed, mainly in the subgrain aggregates on the right-hand side of the map. The white asterisk marks the grain analysed in Fig. 7 ▸(*b*).

As mentioned before, far-field 3DXRD and HEDM data were acquired simultaneously thanks to the semi-transparent near-field detectors. Although reconstructions and indexing were carried out separately with different algorithms, aligning and comparing the results of such simultaneous acquisitions is facilitated by the fact that the measurements refer to the same illuminated volume and scanning conditions. Fig. 8 ▸ compares the HEDM and 3DXRD reconstructions of layer No. 3 in terms of spatial position and crystallographic orientation. The grain size obtained from the far-field data is represented by a black circle centred at the centre-of-mass position of the grain, its radius being proportional to the grain volume. A good visual agreement between the two results can be stated, but significant differences exist in the light-blue region on the right hand side of the map formed by smaller grains and subgrains with close orientations.

**Figure 9:**
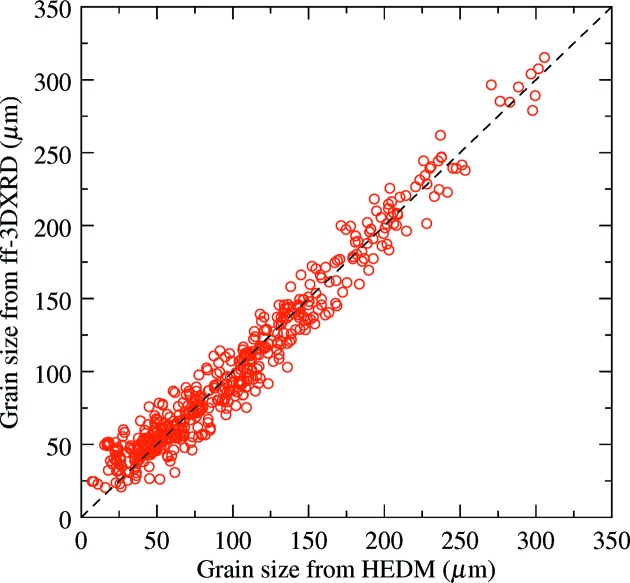
Grain size comparison between HEDM and far-field 3DXRD. Indexing and matching were done separately for seven out of the 14 slices (Nos. 1, 3, 5, 7, 9, 11 and 13). Only far-field grains that were unequivocally matched with HEDM grains have been plotted (347 points).

Grain size was also estimated quantitatively from far-field measurements using the method described by Nervo *et al.* (2014 ▸), which assumes that the grain-to-sample volume ratio is equal to the grain-to-sample ratio of the diffracted intensity. Due to the small and constant height of the beam (about 2 µm), the volume ratio is simplified in the present case to a cross-section ratio. Far-field integrated intensities were first corrected by the structure and Lorentz factors (Poulsen, 2004 ▸) and their average was calculated. The total integrated intensity was considered proportional to the sample cross-sectional area of 1 mm^2^. To facilitate quantitative comparison, the grain shape was considered to be a circle and an equivalent grain diameter was calculated from both 3DXRD and HEDM data. In the latter case, the equivalent grain diameter was calculated as the diameter of the circle having the same area as the real grain in the reconstruction. For selecting grains in the comparison, additional criteria based on grain position (centre-of-mass position obtained from 3DXRD had to be located in the reconstructed HEDM grain) and crystallographic orientations (disorientation angle smaller than 1°) were applied. Fig. 8 ▸ shows about 50 such grains found in layer No. 3. The quantitative results (Fig. 9 ▸) indicate a good agreement for large grains, but this deteriorates for small ones (< 50 µm) where the points lie above the 45° line, indicating that far-field 3DXRD overestimates the true size. The size of the smallest grain identified by far-field 3DXRD is about 25 µm (half the far-field pixel size of 47.5 µm), while the largest grain is 16 times larger with a size of about 325 µm.

## 5. Discussion   

The two near-field diffraction microscopy techniques, DCT and HEDM, discussed mainly in this article have already shown their applicability to numerous problems in materials science, demonstrating the soundness of the retrieved results and indirectly validating the techniques. In terms of the reconstructed grain geometry, DCT has been checked several times *versus* EBSD (Johnson *et al.*, 2008 ▸; Syha *et al.*, 2013 ▸; Lenthe *et al.*, 2015 ▸) as well as *versus* phase contrast tomography (Ludwig, Reischig *et al.*, 2009 ▸). However, all validations performed until recently were done on non-deformed samples such as recrystallized 1070 aluminium alloy (Johnson *et al.*, 2008 ▸) or SrTiO_3_ ceramic (Syha *et al.*, 2013 ▸; Lenthe *et al.*, 2015 ▸). In the present case, an Al–0.3 wt%Mn alloy tensile deformed up to a strain of 1% has been studied. Evidently, validating subgrain structures reconstructed by DCT can still be done *versus* classical EBSD, but the availability of the three-dimensional detector at ID11 (ESRF) naturally suggests that the optimal comparison would be against HEDM, which has already shown its applicability to plastically deformed metals (Li *et al.*, 2012 ▸; Hefferan *et al.*, 2012 ▸). Comparing several HEDM slices with the DCT volume adds statistical significance to the results and preserves the advantage of the nondestructive nature of HEDM and shorter scanning times compared with EBSD, which becomes laborious when coupled with destructive serial sectioning (Lenthe *et al.*, 2015 ▸). However, the average orientation spread in the grains and subgrains of the studied sample (due to a plastic strain of 1%) was small, with both EBSD and HEDM results (Fig. 7 ▸) indicating an IGD distribution with an average of about 0.3°. This is smaller than the disorientation limit of 1° imposed in DCT. Figs. 2 ▸(*a*) and 2 ▸(*b*) show that DCT has captured the majority of subgrain boundaries with disorientations between 1 and 5°, which is a small but important improvement compared with previous results with a disorientation limit of 3° (Syha *et al.*, 2013 ▸).

With the changing beam geometry (from a broad beam in DCT to a line beam in HEDM), small misalignments are introduced between the HEDM and DCT reference frames, which need to be corrected. A general registration method­ology has been developed and implemented to determine the location of two-dimensional HEDM slices in the three-dimensional DCT map. An approach based on disorientation minimization between the two maps was already proposed by Li (2011 ▸) to align successive slightly different HEDM reconstructions, but without considering different acquisition frames or accounting for image distortions. To the best of our knowledge, the type of registration shown here has never been done before in such a general way. The method has also been applied to find the position of the EBSD map in the DCT volume and it worked well even in this general case, characterized by a non-negligible acute angle between the EBSD cut and the DCT *xz* plane, which eliminates the need to match the grains manually (Lenthe *et al.*, 2015 ▸). Based on its general formulation [equations (1)–(5)], the method is potentially applicable to any vector field and can combine both global and local optimization. Considering a constant orientation in the DCT grains, evidently the method is not suitable for treating columnar structures. However, for spatially resolved three-dimensional orientation maps (Viganò *et al.*, 2014 ▸; Lenthe *et al.*, 2015 ▸) it could be used as a standard.

The theoretical approach is based on the minimization of the sum of local disorientations between the pixels in the two-dimensional map and the intersected voxels of the three-dimensional map. The behaviour of the method is determined by the choice of the merit function ϕ(θ). Using a simple identity function would shift grains toward their neighbours with lower disorientations. Therefore, ϕ(θ) as defined in equation (5) will equalize the contribution of higher disorientation angles (θ_*p*_ > 5θ_c_), but these will still have a stronger impact on the merit function than disorientations with θ_*p*_ < 5θ_c_. The choice of θ_c_ = 1° seems reasonable in the light of the IGD spread obtained from HEDM and EBSD (Fig. 7 ▸) and the disorientation values observed in the non-overlapping regions (Fig. 4 ▸). Performing the registration with θ_c_ = 0.2° or θ_c_ = 3° did not significantly change the overlap ratio between DCT and HEDM. However, imposing θ_c_ = 12° led to significant changes in geometric parameters and loss of overlap, indicating the necessity of reducing the influence of large disorientations.

It is important to remark on the similarity between the distribution of the residual θ_*p*_ (Fig. 5 ▸) and that of the IGD obtained from HEDM (Fig. 7 ▸). Indeed, for DCT grains with constant orientation, the orientation variation in the matched HEDM grains will constitute a lower limit for the minimization procedure. This implicitly indicates that the accuracy of the orientation match between DCT and HEDM can not be better than the average disorientation spread of HEDM, which in the present case is about 0.4°. The similarity between the residual θ_*p*_ and the IGD distribution is less good for EBSD, which is certainly influenced by the reduced statistics.

The second aspect of the comparison concerns the spatial resolution of the reconstructions, where the results show an average distance of 4 µm between boundaries identified by DCT and HEDM, and 5.6 µm for DCT and EBSD. The latter value is somewhat larger than the previously reported 1.6–3 µm (Johnson *et al.*, 2008 ▸; Ludwig, Reischig *et al.*, 2009 ▸; Syha *et al.*, 2013 ▸). The largest difference is with regard to the value given by Syha *et al.* (2013 ▸) of 1.6 µm, which was obtained for an undeformed sample with a smaller voxel size of 0.7 µm and a smaller EBSD step of 1 µm. Different sources of error can be pointed out for DCT and HEDM, which are either intrinsic to the techniques or specific to the present study. For DCT, a well known artefact arises from the fact that the back-projected grains are usually undersized, leading to gaps of a few voxels in the reconstruction (Johnson *et al.*, 2008 ▸). The latter are filled in using morphological dilation, which is believed to preserve grain shape but might cause small errors in the final grain boundary position. However, larger errors can be observed when a grain is missing from the reconstruction, as is the case in Fig. 6 ▸, which is probably due to neighbouring grains with close disorientations of about 1°. For the present measurements, DCT is also expected to be influenced by the intragranular orientation spread induced by plastic deformation, which is inconsistent with the parallel-beam assumption of back-projection (single orientation per grain). The present analysis has shown that DCT failed to detect 60% of the subgrain boundaries identified by HEDM which had disorientations between 1 and 5°. Therefore, a disorientation of 1° can be considered a limit of the current DCT code, and more sophisticated six-dimensional reconstruction algorithms (Viganò *et al.*, 2014 ▸) are needed to cope with real deformed structures.

Regarding HEDM, the grain shapes are mainly determined by the intensity binarization algorithms prior to the reconstruction. The low confidence values obtained here, which may be related to the low signal-to-noise ratio of the measurements and the misalignment of the rotation axis, imply a smaller number of Bragg peaks used for the reconstruction. This leads to reduced resolution of grain cross-section shapes, as well as to reduced orientation resolution (typically quoted as 0.1° for the standard data collection scheme). Besides the spatial resolution being assessed by Euclidean distance mapping in two-dimensions, larger errors arise when a grain boundary is cut at a small angle, overestimating the actual difference between the microstructures. Therefore, the agreement between HEDM, DCT and EBSD seen here should be interpreted as giving an upper bound on the measurement precision of HEDM and DCT. The average interboundary distance of 4 µm corresponds to about 4% of the average grain size and might represent a relative grain volume difference of about 12% (considering spherical grains). This rough estimate was confirmed by a direct comparison between DCT and HEDM grain volumes (accumulated over the 14 slices), which gave a relative volume difference of about 10%.

It is instructive to discuss the diffraction geometry used for the present HEDM measurements. The semi-transparent ‘three-dimensional detector’ (Poulsen *et al.*, 2010 ▸) allows the simultaneous recording of diffraction peaks by three detectors, which has several advantages compared with the classical set up (Suter *et al.*, 2006 ▸). First of all, thanks to the two tightly fixed near-field detectors (making up the three-dimensional detector), the HEDM acquisition time can potentially be reduced, allowing a more detailed characterization for a given amount of beamtime. However, the exposure time should be adjusted carefully in order to avoid spot loss on the second near-field detector (of 4.5 µm pixel size), which in the present construction is less efficient than the first one (1.5 µm pixel size). Secondly, the extra peaks recorded with the far-field detector can be more readily used to find and index the grains in a given slice. This information can be exploited to speed up the indexing of single pixels in the HEDM map, which could lead to a considerable acceleration of the HEDM reconstruction.

## 6. Conclusions   

A direct comparison of the grain shape and lattice orientation obtained with DCT and HEDM, two near-field diffraction microscopy techniques, has been presented. Accurate comparison required the development of a novel registration method based on minimizing the local disorientation between DCT and HEDM maps. It has been shown that DCT can detect subgrain boundaries with disorientations as low as 1°, but this value should be considered a lower limit for the current DCT code. HEDM and DCT grain boundaries are on average 4 µm apart from each other, which represents a relative grain volume difference of about 10% in the case of the present structure with an average grain size of about 100 µm.

The agreement between DCT and EBSD was found less good than in previous works, also suggesting that a different DCT algorithm is necessary for the reconstruction of deformed microstructures. On the other hand, intragranular disorientation distributions obtained by HEDM and ESBD have shown an excellent agreement.

The comparison between HEDM and far-field 3DXRD showed that equivalent grain diameters determined from the integrated intensity of diffraction spots measured on a far-field detector can be highly inaccurate (with relative errors of more than 100%), which limits the application of the latter method to *in situ* studies targeting grain volume evolution.
